# *Passiflora incarnata* in Neuropsychiatric Disorders—A Systematic Review

**DOI:** 10.3390/nu12123894

**Published:** 2020-12-19

**Authors:** Katarzyna Janda, Karolina Wojtkowska, Karolina Jakubczyk, Justyna Antoniewicz, Karolina Skonieczna-Żydecka

**Affiliations:** Department of Human Nutrition and Metabolomics, Pomeranian Medical University in Szczecin, 71-460 Szczecin, Poland; Katarzyna.Janda@pum.edu.pl (K.J.); lottecharlotte23@gmail.com (K.W.); kaldunskajustyna@gmail.com (J.A.); karzyd@pum.edu.pl (K.S.-Ż.)

**Keywords:** *Passiflora incarnata*, neuropsychiatric disorders, stress, anxiety, depression

## Abstract

Background: Stress is a natural response of the body, induced by factors of a physical (hunger, thirst, and infection) and/or psychological (perceived threat, anxiety, or concern) nature. Chronic, long-term stress may cause problems with sleep, concentration, and memory, as well as affective disorders. The passionflower (*Passiflora incarnata*) is a perennial plant with documented therapeutic properties. The literature data suggest that the passionflower itself, as well as its preparations, helps reduce stress and can therefore be helpful in the treatment of insomnia, anxiety, and depression. The objective of this systematic review was to evaluate *Passiflora incarnata* in terms of its neuropsychiatric effects. Methods: The scientific databases PubMed, ClinTrials.gov, and Embase were searched up to 22 October 2019. The search identified randomized clinical trials describing the effects of *Passiflora incarnata* in neuropsychiatric disorders. Results: The systematic review included nine clinical trials. The duration of the studies included in the analysis varied widely, from one day up to 30 days. Study participants were no less than 18 years old. In each of the papers, the effects of passionflower were measured by using a number of different tests and scales. The majority of studies reported reduced anxiety levels following the administration of *Passiflora incarnata* preparations, with the effect less evident in people with mild anxiety symptoms. No adverse effects, including memory loss or collapse of psychometric functions, were observed. Conclusion: *Passiflora incarnata* may be helpful in treating some symptoms in neuropsychiatric patients.

## 1. Introduction

The passionflower (*Passiflora incarnata* L.) is a perennial plant which that can grow up to 10 m, with egg-shaped edible fruit. The low-calorie fruit (41–53 kcal/100 g) is a rich source of vitamins A, C, B1, and B2, as well as calcium, phosphorus, and iron. The species is native to South America, Australia, and South East Asia, and today is cultivated to source raw material for pharmaceutical use [1]. *Passiflora incarnata* is one of the best-documented species of the *Passiflora* genus with therapeutic properties. The aerial parts of the plant, flowers, and fruits are used for medicinal purposes. They are credited with anthelmintic, antispasmodic, and anxiolytic effects. The passionflower is also used as a remedy for burns, diarrhea, painful menstruation, hemorrhoids, in neurotic disorders, insomnia, to treat morphine dependence, and can be helpful in convulsions or neuralgia, too. *Passiflora incarnata* is a source of alkaloids, phenolic compounds, flavonoid, and cyanogenic glycosides. The primary phytochemicals found in the passionflower are flavonoids (apigenin, luteolin, quercetin, and kaempferol) and flavonoid glycosides (vitexin, isovitexin, orientin, and isoorientin) [1,2]. The species has the highest overall isovitexin content [3]. On 25 March 2014, the European Medicines Agency published a herbal monograph on *Passiflora incarnata*, thus recognizing its status as a medicinal product [4]. Clinical trials found no threats to human health in relation to the use of *Passiflora incarnata* [5,6].

Stress is a natural response of the body. It can be induced by factors of a physical (hunger, thirst, infection) and/or psychological (perceived threat, anxiety or concern) nature—namely stressors. Stress has been linked to cellular inflammation. Physiologically, the body’s response to stress causes an immediate activation of the adrenergic system and the sympathetic–adrenomedullary axis (SAM axis), followed by the hypothalamic–pituitary–adrenal axis (HPA axis). Chronic, long-term stress is a pathological condition, which may impair concentration and memory, as well as lead to affective disorders, such as depression, schizophrenia, and the post-traumatic stress disorder [7]. *Passiflora incarnata* is one of the herbal remedies used to alleviate the effects of stress [2]. A rat study demonstrated that long-term use of passionflower was correlated with reduced stress levels and, consequently, increased motivation to act and improved motor activity [8]. The beneficial effects of passionflower on memory function have also been confirmed [9]. The use of *P. incarnata* in people with chronic insomnia may produce a therapeutic effect in the management of sleep disorders, memory loss, and degenerative brain diseases. *Passiflora* may be helpful in the treatment of insomnia, through its sedative action, as a result of which the person experiencing difficulty sleeping will be more likely to get to sleep [10]. *Passiflora* demonstrates positive effects in episodes of anxiety, restlessness, sleeplessness, and in depressive states [11].

The objective of this systematic review was to evaluate the efficacy of *Passiflora incarnata* preparations in the treatment of neuropsychiatric disorders. The systematic review included randomized controlled trials (RCT) which investigated the relationship between the use of *Passiflora incarnata* and a range of disorders of the nervous system.

## 2. Materials and Methods

### 2.1. Search Strategy, Inclusion Criteria

At least two independent authors (K.W., J.A. and K.S.Z.) searched PubMed/MEDLINE/Embase, from database inception until 22 October 2019, without language restrictions. Randomized clinical trials have been found to describe the effect of the use of passion flower in neuropsychiatric disorders. The following search string in Pub Med was used: (“passiflora” OR “passion fruit” OR “passion” OR “passion flower”) AND (anxiety OR depression OR insomnia OR somatoform); Embase (“passiflora”/exp OR “passiflora”) AND (“depression”/exp OR “central depression” OR “clinical depression” OR “depression” OR “depressive disease” OR “depressive disorder” OR “depressive episode” OR “depressive illness” OR “depressive personality disorder” OR “depressive state” OR “depressive symptom” OR “depressive syndrome” OR “mental depression” OR “parental depression” OR “anxiety disorder”/exp OR “anxiety disorder” OR “anxiety disorders” OR “insomnia”/exp OR “agrypnia” OR “hyposomnia” OR “insomnia” OR “sleep initiation and maintenance disorders” OR “sleeplessness” OR” somatoform disorder”/exp OR “somatoform disorder” OR “somatoform disorders”), oraz ClinTrials.gov (*Passiflora*).

We utilized the following inclusion criteria: (i) original studies, (ii) studies with access to full text, (iii) studies in which the treatment included any products (supplements, tinctures, extracts, infusions, raw materials, etc.) containing *Passiflora incarnata*, (iv) presence of meta-analytical data (change score/endpoint) on psychiatric symptoms in the process of each neuropsychiatric disease, and (v) studies carried out in humans. Exclusion criteria were as follows: (i) intervention with products containing other psychoactive substances; (ii) meta-analyses, systematic reviews, and review works.

### 2.2. Data Abstraction

Data for country in which the study was conducted, information about the sponsors, type of blindness, duration of the study, and main purpose of the study, as well as the name of the preparation used during therapy, were extracted. During data abstraction, detailed data on the studied population were looked for, i.e., the average age and standard deviation of studied persons, the number and percentage of men participating in the study, and the number of people randomized to the study. Data extraction was performed based on the guidelines contained in the Preferred Reporting Items for Systematic Reviews and Meta-Analyses (PRISMA) protocol, but with no study protocol registration. If data were missing, authors were contacted via email, to ask for additional information. Inconsistencies were resolved by consensus, with the corresponding author being involved. The results that were compared in the systematic review involved various scales and tests, such as the Hamilton Rating Scale for Depression (HRSD), Visual Analogue Scale (VAS), Numerical Rating Scale (NRS), Observers Assessment of Alertness and Sedation Scale (OAA/S), Corah’s Dental Anxiety Scale, Revised (DAS-R), Ramsey Scale, Digit symbol substitution test (DSST), Concentration Endurance Test, (The d2 test), Memory test, Continuous Performance Task/Test (CPT), Trieger Dot Test (TDT), Perceptive Accuracy Test (PAT), Finger Tapping Test (FTT), and State-Trait Anxiety Inventory (STAI-S, STAI-T). Data from charts and figures were extracted by means of WebPlotDigitizer software (https://automeris.io/WebPlotDigitizer/) in order to detect the risk of bias, the Cochrane Collaboration’s tool for assessing risk of bias was used.

### 2.3. Outcomes

The primary outcome was to evaluate the effects of a *Passiflora incarnata* on neuropsychiatric symptoms, namely depressive/anxious phenotype and reactivity to stress. Co-secondary outcomes were insomnia, somatoform and psychomotor functions, sedation, and nervous restlessness.

## 3. Results

### 3.1. Search Results

**Figure 1:**
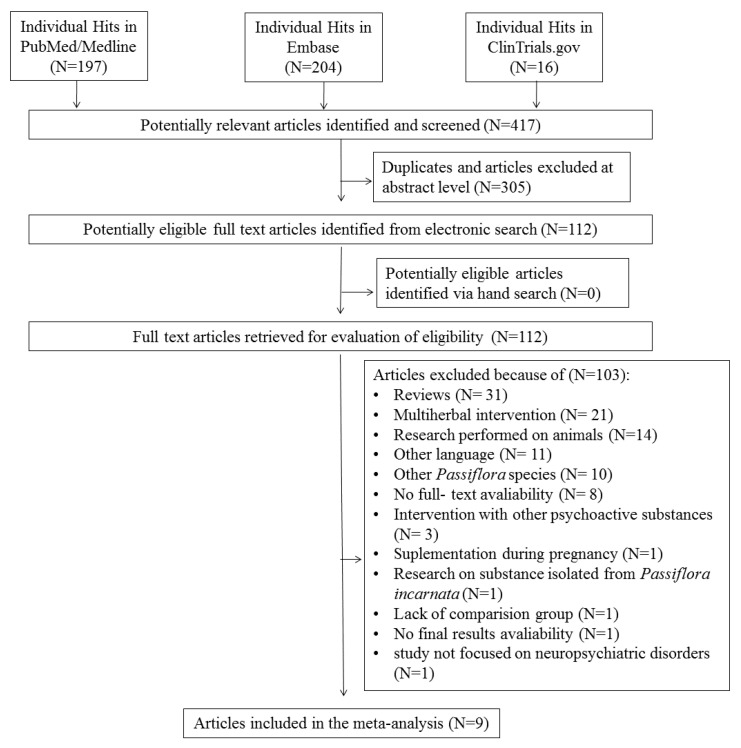
Study flowchart.

The first search in PubMed and Embase databases resulted in 417 hits. Among them, 305 studies were excluded as duplicates and/or after evaluation at the title/abstract level. After excluding 305 studies, 112 full-text articles were eventually reviewed, 103 of which were excluded due to the failure to meet previously established inclusion criteria. The main reasons for exclusion were as follows: review (*n* = 31), intervention with multi-herbal preparations (*n* = 21), an article published in a language other than Polish or English (*n* = 11), lack of access to full text (*n* = 8), intervention with other psychoactive substances (*n* = 3), supplementation during pregnancy (*n* = 1), review of the substance isolated from *Passiflora incarnata* (*n* = 1), lack of availability of final results (*n* = 1), a study in which a comparison group was missing (*n* = 1), and a study that did not focus on neuropsychiatric disorders (*n* = 1). Finally, nine studies were included in the systematic review. The scheme of searching databases is included in Figure 1.

### 3.2. Study Characteristics

Nine studies carried out between 2017 and 2019 were included in this systematic review.

The objective of this systematic review was to evaluate *Passiflora incarnata* in terms of its neuropsychiatric effects. More than half of the studies (five) were carried out in Iran, and the others in Brazil, Turkey, Germany, and Australia. The vast majority of study participants was healthy [6,12,13,14,15,16,17,18]. There was only one study [19] in which the participants had a diagnosis of Generalized Anxiety Disorder (GAD). The duration of the studies included in the analysis varied widely—from one day up to 30 days. Predominantly, the aim of the reviewed papers (*n* = 4) was to assess the effects of passionflower use on the anxiety experienced by patients during spinal anesthesia, dental procedures, or surgery. Studies investigating the effects of *P. incarnata* administration on sleep quality and cognitive functions were also included in the analysis. The majority of the analyzed studies (*n* = 7) were double-blind (DB), with one cross-over study. The other trials (*n* = 2) were single-blind (SB), and one (*n* = 1) was a cross-over study. In all the papers included in the review, the participants were no less than 18 years old. Details of the studies included in the systematic review are presented in Table 1.

### 3.3. Effect of Passiflora Treatments on Neuropsychiatric Parameters

The systematic review included nine studies. In each of the works, different criteria were taken into account: the Hamilton Rating Scale for Depression (HRSD), Observers Assessment of Alertness and Sedation Scale (OAA/S), Corah’s Dental Anxiety Scale, Revised (DAS-R), Ramsey Scale, Digit symbol substitution test (DSST), Continuous Performance Task/Test (CPT), Trieger Dot Test (TDT), Perceptive Accuracy Test (PAT), Finger Tapping Test (FTT), State-Trait Anxiety Inventory (STAI), Visual Analogue Scale (VAS), memory test, and Concentration Endurance Test (d2 test). The results of the use of the *Passiflora* preparations are presented in Table 2 and Table 3.

The Hamilton Depression (HAM-D) Scale is a numerical scale consisting of 21 points (or 17 points in some cases). It is a tool used in general psychiatry, to assess the diagnosis of depression and to clinically evaluate the use of antidepressants. The HAM-D score level of depression is as follows: 10–13, mild; 14–17, mild to moderate; and >17, moderate to severe [20]. The numerical and analog-visual scales belong to pain scales with scores ranging from 0 to 10, with score 0 reflecting no pain and 10 the strongest imaginable pain. The four-stage OAA/S scale is a useful tool in assessing the awareness of patients who have received midazolam. The scale has been used for sedation-related drugs, to assess a person’s level of sedation, since the 1990s [21]. The Corah Dental Anxiety Scale has been used since the 1970s [22]. It consists of four items (questions) on dental anxiety. Each answer is scored from 1 to 5. A total of 20 points can be obtained, with a score above 15 suggesting the presence of a dental phobia [23]. The Corah scale is another Dental Anxiety Scale which can be used in children [24]. The Ramsey scale is one of the most widely used scales to assess the level of sedation. Scores are recorded from 0 to 6, with 0 being conscious and 6 being deeply coma [25]. The DSST test is primarily used in clinical neuropsychology. Initially, it helped scientists to understand how a person learns. Currently, it is used in the assessment of cognitive disorders, such as schizophrenia and major depression [26]. The concentration strength test concerns the diagnosis in the context of concentration, as well as the perception and possible correction of errors [27]. The memory test mainly focuses on visual memory. It informs about possible alterations within the central nervous system, and it can also be used to assess the attention/concentration disorders. The continuous exercise test is a neuropsychological test that is used in the diagnosis of ADHD and epilepsy, as well as in patients with brain damage. It focuses mainly on the patient’s constant attention, while allowing us to measure the degree of impulsiveness during the test. It helps to collect quantitative data about the patient [28,29]. Measurement of perceptual-motor skills is performed by using the Trieger point tests (e.g., TDT) or the exact perception tests (e.g., PAT) [30,31]. TDT is also a useful tool for assessing the level of anesthesia and recovery [32]. The finger tapping test is one of the standard neuropsychological assessments that examines motor functioning, specifically, motor speed and lateralized coordination. The inventory of the state and trait of anxiety allows the assessment of the severity of anxiety and its characteristics [33].

### 3.4. Risk of Bias Assessment

The bias analysis showed that the three studies were of low quality and received less than 5 points in the risk of bias (ROB) evaluation [13,16,17]. In the last six [6,12,14,15,18,19], the number of points was higher than 5. The average number of points in all studies is 5.66 The results of the bias risk analysis are presented in Table 4.

## 4. Discussion

Neuropsychiatric disorders, such as schizophrenia, bipolar affective disorder, major depressive disorder, and attention-deficit hyperactivity disorder, are a common and, regrettably, increasingly prevalent problem. Around the world, depression affects some 322 million people, while 264 million live with anxiety [34]. Mild dysfunctions of the nervous system can be treated with psychotherapy, but more severe disorders require pharmacological treatment alongside therapy [35]. In the past year, the academic interest in these disorders has been growing due to the COVID-19 pandemic and the related upsurge in anxiety and depression [36]. Pharmacotherapy is effective, but, at the same time, it carries the risk of side effects and dependence [37]. Hence, the search for herbal remedies for neuropsychiatric disorders continues to go on [38]. *Passiflora incarnata* is a perennial plant containing precious phytochemicals with health-promoting properties. The most important among them would appear to be chrysin, due to its neuroprotective effects [39]. The systematic review method used in this study made it possible to assess the efficacy of passionflower with respect to neurological disorders, by synthesizing the results of nine clinical trials included herein. This is the first systematic review evaluating the effects of *Passiflora incarnata* in neuropsychiatric disorders.

Nine clinical trials were included in this paper. The reviewed studies analyzed the effects of passionflower preparations on anxiety levels experienced by patients during medical interventions, including spinal anesthesia, dental procedures, or surgery, as well as on sleep quality and cognitive functions. In eight papers, the study subjects were healthy, and, in one, *P. incarnata* was given to patients with a diagnosis of Generalized Anxiety Disorder (GAD). Various commercial products containing passionflower preparations were administered in the analyzed trials, including drops, tablets, and syrup. Detailed information, including the type of preparation and dosage, is presented in Table 1.

Akhondzadeh et al. [12], in their study of people with Generalized Anxiety Disorder (GAD), compared the effects of passionflower extract with oxazepam over a period of 28 days. To this end, they used the Hamilton Rating Scale for Depression. Study participants receiving either passionflower extract (45 drops/day) or oxazepam were evaluated each day, prior to, during, and after taking the relevant substance (Table 2). The authors demonstrated that there were no significant differences between taking passionflower vs. oxazepam, and the former did not cause an impairment of job performance in the subjects. A follow-up large-scale trial was recommended.

Aslanargun et al. [13] investigated the effects of administering passionflower syrup (700 mg/5 mL, 30 min before anesthesia) on anxiety, psychomotor function, sedation, and hemodynamics in patients before spinal anesthesia. The effects of passionflower were examined in awake patients after surgery (Table 2). The authors demonstrated that *P. incarnata* significantly contributed to reducing preoperative anxiety. Even though it was reported that psychomotor functions were impaired 30 min after extubation, the preoperative values were restored by 90 min. Side effects, including cutaneous vasculitis, urticaria, asthma, or rhinitis, were not observed. Hemodynamic parameters did not change after the administration of *Passiflora*, as compared to the placebo. Additional advantages included the lack of intraoperative sedation and respiratory depression. According to the authors, *P. incarnata* is a safe and effective anxiolytic which can be used before spinal anesthesia.

The objective of the study by Azimaraghi et al. [14] was to compare the efficacy of passionflower and oxazepam in reducing patients’ preoperative anxiety. The authors demonstrated that patients who were given *Passiflora* tablets (500 mg for premedication) had lower preoperative anxiety levels, as compared to the group receiving oxazepam, and the effects of both medications on postoperative psychomotor function were similar. Recovery time was, likewise, comparable in both groups (Table 2). The authors suggest that *Passiflora incarnata* is safe and definitely more effective for reducing preoperative anxiety in comparison to oxazepam. They also point out that it can be included in the treatment of preoperative anxiety in children and adolescents.

To compare the anxiolytic action of *Passiflora incarnata* with that of midazolam, Dantas et al. [15] employed an experimental model involving bilateral extraction of the mandibular third molars. The participants received 15 mg of midazolam (one pill) or 260 mg of *Passiflora incarnata* (one pill) administered orally 30 min before the start of the surgical procedure. In a cross-over design, participants were randomly assigned an extraction side (right or left) and a protocol (midazolam or *Passiflora*) at the first procedure. The researcher delivered the drugs to the participants, in encoded form, as “Protocol 1” (midazolam) or “Protocol 2” (*Passiflora*). The patients were asked to specify whether they felt calm, a little anxious, very anxious, or so anxious that they felt bad. Detailed results are presented in Table 3. Higher levels of anxiety were observed in women than in men. The anxiolytic action of both substances used in the study was similar. Among the participants in the midazolam group, 20% reported they did not remember anything, while none of the patients receiving passionflower reported such an experience. In terms of adverse effects, somnolence was reported by 82.5% of the participants who received midazolam, and 50% in the Passiflora group. When given the choice, 52% of the participants would opt for surgery with midazolam, and 27.5% for the *P. incarnata* treatment, while the remainder found no difference between these interventions. The authors suggest that the higher preference for midazolam among the participants was related to the effect of amnesia, which prevented the formation of negative memories.

Dimpfel et al. [16] evaluated the effects of NEURAPAS^®^ (192 mg of *P. incarnata* extract) on brain electric activity. Electroencephalogram (EEG) recordings were made at 30 min and 1.5, 3, and 4 h after administering the preparation. EEG tests were performed during the Concentration Endurance Test, memory test, and Continuous Performance Task/Test. Results are presented in Table 2. No differences were observed in the analyzed psychometric parameters between NEURAPAS^®^ vs. placebo. Sixteen participants receiving NEURAPAS^®^ obtained higher values in the Continuous Performance Task/Test. The analysis of neurophysiological alterations after taking NEURAPAS^®^ revealed frequency changes in EEG that were similar to those of sedative and antidepressant medications, without impairing cognitive function.

The objective of the study by Kaviani et al. [17] was to evaluate the effects of the passionflower extract on anxiety levels in psychiatrically healthy patients undergoing dental treatment. No differences were observed in mean anxiety scores before taking the medication (Table 2). The authors emphasize their important finding of very effective anxiety-reducing action of the passionflower. They also acknowledge the need for further research on *P. incarnata*.

Movafegh et al. [6] investigated the effects of passionflower (500 mg) on anxiety in surgery patients. The results of their tests are presented in Table 2. The authors conclude that *P. incarnata* at 500 mg/day provides a safe and effective anxiolytic effect, without impairing psychomotor function. At the same time, they strongly stress that their sample was too small (*n* = 60) and urge that research should be continued with a larger group.

Ngan and Conduit [18] analyzed the effects of *Passiflora incarnata* herbal tea on sleep quality over a period of seven days, as measured by using sleep diaries and polysomnography. The participants drank 250 mL of the herbal tea once a day, in the evening (to avoid the sedative effect during the day), and the measurements were performed in the morning, upon rising. The State-Trait Anxiety Inventory (STAI-S) was used to evaluate the efficacy of passionflower infusions, but the results were not included in the report from the study. An attempt was made to contact the authors to obtain their results, but there was no answer. In terms of subjective sleep-quality parameters, a significant improvement of the reported sleep quality (SQ) was observed in the *Passiflora* treatment, with a mean increase of 5.2%, compared to the placebo. The authors highlight that passionflower may have a limited impact on sleep quality in people with low anxiety levels. Their findings may also have been affected by the long interval between drinking the herbal tea and the measurement of anxiety.

The objective of the study by Rokhtabnak et al. [19] was to compare the effects of premedication with melatonin vs. *Passiflora incarnata* on the cognitive function in adult patients undergoing elective surgery. No significant differences in pain scores were observed between the groups, either before or after surgery. The Digital Symbol Substitution Test revealed better postoperative results for melatonin than *Passiflora*. Both groups showed reduced anxiety and increased sedation scores in the Ramsey test. Detailed results are presented in Table 2. The authors report positive effects of both interventions on reducing patient anxiety.

*Passiflora incarnata* is important in herbal medicine for treating anxiety or nervousness, Generalized Anxiety Disorder (GAD), symptoms of opiate withdrawal, insomnia, neuralgia, convulsion, spasmodic asthma, ADHD, palpitations, cardiac rhythm abnormalities, hypertension, sexual dysfunction, and menopause. However, the mechanism of action is still under discussion. Despite gaps in our understanding of neurophysiological processes, it is increasingly being recognized that dysfunction of the GABA system is implicated in many neuropsychiatric conditions, including anxiety and depressive disorders. Therefore, the in vitro effects of a dry extract of *P. incarnata* on the GABA system were investigated. The extract inhibited [3H]-GABA uptake into rat cortical synaptosomes but had no effect on GABA release and GABA transaminase activity. *P. incarnata* inhibited concentration dependently on the binding of [3H]-SR95531 to GABAA-receptors and of [3H]-CGP 54626 to GABAB-receptors. Using the [35S]-GTPγS binding assay, *Passiflora* could be classified as an antagonist of the GABAB receptor. In contrast, the ethanol- and the benzodiazepine-site of the GABAA-receptor were not affected by this extract. In conclusion, the first evidence was shown that numerous pharmacological effects of *P. incarnata* are mediated via modulation of the GABA system, including affinity to GABAA and GABAB receptors, and effects on GABA uptake [40]. Aman et al. carried out research on mice which indicated that *P. incarnata* may be useful in treating neuropathic pain. The authors suggested that these properties may result from underlying opioid and GABA-ergic mechanisms, but also pointed to the potential involvement of oleamid-based cannabimimetics [41]. The mechanism of action cannot, at present, be regarded as clarified; however, more recent studies imply that the anxiolytic effects may be mediated via modulation of the GABA system [42,43,44]

This systematic review has some limitations. First, there are few studies on the effects of *Passiflora incarnata* in neuropsychiatric disorders. Taking into account the inclusion and exclusion criteria, only nine works were qualified for the present synthesis. Secondly, almost all authors postulate to continue research in large-scale populations. In the analyzed publications, the study groups ranged from only 16 to 128 persons. Moreover, it has been proposed to include populations of different ethnicities in continuing the research. Due to the high heterogeneity, it was impossible to perform a meta-analysis, which further suggests that research in this area should be continued.

Another limitation may be the lack of information on which part of the plant was used in the research. Traditional medicine uses the leaves, stamps, seeds, and flowers (aerial parts) of *P. incarnate* [4]. Unfortunately, the authors usually do not specify in their publications what part of the plant was used by them. Ngan and Conduit [18] indicated that they used leaves, stamps, seeds, and flowers. Perhaps all the others also used all the aboveground parts as a mixture. It seems advisable, therefore, for the authors to indicate what part of the plant they used in their research, as this may be relevant for the interpretation of the results and discussion.

In conclusion, the authors of the works included in this systematic review all agree that *Passiflora incarnata* may be an effective, cheap, and safe drug used in counteracting at least some of the symptoms of neuropsychiatric origin. At the same time, they indicate the advisability of continuing research on a large population of people from various geographical regions.

## 5. Conclusions

Passionflower has the potential to alleviate some symptoms of neuropsychiatric origin. No adverse effects, including memory loss or collapse of psychometric functions, have been linked to passionflower administration. The anti-anxiety effect of *Passiflora incarnata* is comparable to drugs such as oxazepam or midazolam. Consequently, it seems to be an effective and safe pharmaceutical to reduce stress reactivity, insomnia, anxiety, and depression-like behaviors.

## Figures and Tables

**Table 1:** Characteristics of studies included in the systematic review.

Reference/Year/Country	Description of Treatment				Characteristics of The Intervention and of the Study Group					
	Study Objective	Blinding	Duration of Administration (Days)	ROB *	Commercial Name of Product Containing *P. Incarnata*	*Passiflora* Dose	Comparator	*n* Total Randomized/Analyzed	Age Years (Mean)	Males (%)
Akhondzadeh et al., 2001 (Iran) [12]	Comparative efficacy of *Passiflora incarnata* extract with oxazepam in the treatment of systemic anxiety disorder	DB	28	5	Passipay (Iran, Darouk)	45 drops/day	Placebo + oxazepam (30 mg/day)	36/32	19–47 #	44.4
Aslanargun et al., 2012 (Turkey) [13]	Effects of preoperative oral administration of *Passiflora incarnata* on anxiety, psychomotor functions, sedation and hemodynamics in patients undergoing spinal anesthesia	DB	1	4	*Passiflora* Syrup (Sandoz, Kocaeli, Turkey)	700 mg/5 mL	Placebo	60/60	25–55 #	86.6
Azimaraghi et al., 2017 (Iran) [14]	The efficacy of *Passiflora incarnata* to reduce preoperative anxiety in comparison to oxazepam	DB	NA	7	Passipy™ (Iran Darouk)	500 mg	Oxazepam (10 mg)	128/128	36.77	57.8
Dantas et al., 2017 (Brazil) [15]	The influence of *Passiflora incarnata* and midazolam on the control of anxiety in patients exposed to the extraction of the third mandibular molar tooth	DB, CO	15-30	7	*Passiflora incarnata*	260 mg	Midazolam (15 mg)	40/40	23.94	32.5
Dimpfel et al., 2011 (Germany) [16]	Explanation of the effectiveness of the preparation by analysis of current density (CFD) of brain activity in the presence of various mental challenges	SB, CO	1	4	NEURAPAS^®^	192 mg *of P. incarnata* extract	Placebo	16/16	47.85	50
Kaviani et al., 2013 (Iran) [17]	Determining the effectiveness of passion flower application in reducing anxiety during dental procedures	SB	2	4	Passipay (Iran, Darouk)	40 drops/day	Placebo	63/63	34.07	38.1
Kaviani et al., 2013 (Iran) [17]	Determining the effectiveness of passion flower application in reducing anxiety during dental procedures	SB	2	4	Passipay (Iran, Darouk)	40 drops/day	Negative group	63/63	34.07	38.1
Movafegh et al., 2008 (Iran) [6]	Effectiveness of *Passiflora* in reducing preoperative anxiety	DB	1	7	Passipy™ (Iran Darouk)	500 mg	Placebo	60/60	31.85	50
Ngan and Conduit, 2011 (Australia) [18]	To test the effectiveness of the *Passiflora* infusion on human sleep, measured by means of sleep logs approved by polysomnography	DB	22	6	Tea bags (Hilde Hemmes’ HerbalSupplies Pty Ltd.; SA, Australia)	infusion (2 g in 250 mL water; concentration 0.8%)	Placebo	41/41	22.73	34.1
Rokhtabnak et al., 2017 (Iran) [19]	Effects of *Passiflora incarnata* and melatonin on cognitive functions and sedative effect without causing cognitive disturbance	DB	1	7	*Passiflora incarnata*	1000 mg (prepared and packed by Department of Pharmacy, Shahid Beheshti University of Medical Sciences, Iran)	Melatonin (6 mg)	64/52	20–60 #	44.2

Notes: #—range; *—the risk of bias (ROB), shown in numbers; DB—double blind; SB—single blind; CO—cross-over; NA—not available.

**Table 2:** Results of the systematic review.

Reference/Year/Country	Comparator	Control Group		Tested Group		Conclusions
		Baseline Data	Endpoint Data	Baseline Data	Endpoint Data	
Akhondzadeh et al., 2001 (Iran) [12]	Placebo + oxazepam (30 mg/day)	Hamilton scale: 19.74 ± 0.83	Hamilton scale: 5.1 ± 1.28	Hamilton scale: 19.74 ± 0.83	Hamilton scale: 5.5 ± 0.75	*Passiflora* extract is effective in the treatment of systemic anxiety; additionally, there is a low incidence of impairment of work efficiency with *Passiflora* extract, as compared to oxazepam. There is a need for further research on the use of *Passiflora* in the treatment of systemic anxiety.
Aslanargun et al., 2012 (Turkey) [13]	Placebo	STAI-S: 34.8 ± 8.4; STAI-T: 35.3 ± 8.3; PAT: 95.2 ± 16.4; FTT: 72.3 ± 14.1; OAA/S: 5 ± 0; NRS: 6.6 ± 1; TDTmm: 0.8 ± 0.2; TDTnr: 0.9 ± 0.7; DSST: 30.8 ± 5;	STAI-S: 36.6 ± 7.6; STAI-T: 38.1 ± 9.2; PAT: 99.1 ± 1.4; FTT: 72.3 ± 13.1; OAA/S: 5 ± 0.15; NRS: 6.1 ± 1.3; TDTmm:1.1 ± 0.3; TDTnr: 1.2 ± 1; DSST: 29.1± 4.8;	STAI-S: 36.4 ± 10.9; STAI-T: 32.5 ± 9.5; PAT: 98.0 ± 2.6; FTT:67.4 ± 18.9; OAA/S: 5 ± 0; NRS:7.6 ± 0.9; TDTmm: 0.9 ± 0.2; TDTnr: 0.8 ± 0.8; DSST: 31.1 ± 5.1;	STAI-S: 35.7 ± 10.8; STAI-T: 33.4 ± 8.7; PAT: 99.1 ± 1.7; FTT: 67.6 ± 19.8; OAA/S: 5 ± 0.15; NRS: 4.4 ± 1.2; TDTmm: 1.2 ± 0.4; TDTnr: 1 ± 0.9; DSST: 28.6 ± 5;	Preoperative oral administration of 700 mg/5 mL of *Passiflora* water extract reduces the level of anxiety in patients before spinal anesthesia without changing their sedation level, psychomotor function test results, or hemodynamics.
Azimaraghi et al., 2017 (Iran) [14]	Oxazepam (10 mg)	NRS: 6.6 ± 1; TDTmm: 0.8 ± 0.2; TDTnr: 0.9 ± 0.7; DSST: 30.8 ± 5;	NRS: 6.1 ± 1.3; TDTmm: 1.1 ± 0.3; TDTnr: 1.2 ± 1; DSST: 29.1 ± 4.8;	NRS: 7.6 ± 0.9; TDTmm: 0.9 ± 0.2; TDTnr: 0.8 ± 0.8; DSST: 31.1 ± 5.1;	NRS: 4.4 ± 1.2; TDTmm: 1.2 ± 0.4; TDTnr: 1 ± 0.9; DSST: 28.6 ± 5;	In outpatient surgery, oral administration of *Passiflora* as a premedication reduces preoperative anxiety with comparable dysfunction of psychomotor functions, as compared to preoperative oral intake of oxazepam.
Dimpfel et al., 2011 (Germany) [16]	Midazolam (15 mg)	d2 test: 12.32 ± 4.02; memory test: 10.77 ± 3.98; ccCPT: 6.57 ± 6.17;	d2 test: 13.59 ± 3.77; memory test: 11.51 ± 3.74; CPT: 6.87 ± 7.3;	d2 test: 12.14 ± 3.06; memory test: 11.37 ± 3.64; CPT: 5.59 ± 5.85;	d2 test: 13.53 ± 3.13; memory test: 11.95 ± 3.65; CPT: 7.86 ± 5.76;	Analysis of neurophysiological changes after NEURAPAS^®^ intake showed similarity of changes compared to sedatives and antidepressants, in EEG, without cognitive function impairment.
Kaviani et al., 2013 (Iran) [17]	Placebo	Corah DAS-R: 12 ± 2.66;	Corah DAS-R: 10.52 ± 2.11;	Corah DAS-R: 13.09 ± 2.42;	Corah DAS-R: 8.47 ± 2.08;	The serving of the passion flower as a premeditation is effective in reducing anxiety. Further trials with more people are needed to confirm the results.
	Placebo	Corah DAS-R: 11.66 ± 2.39	Corah DAS-R: 11.23 ± 2.34	Corah DAS-R: 13.09 ± 2.42;	Corah DAS-R: 8.47 ± 2.08;	
Movafegh et al., 2008 (Iran) [6]	Negative group	NRS: 5.1 ± 2; TDTmm: 0.6 ± 1; TDTnr: 0.8 ± 0.9; DSST: 24.3 ± 6.2;	NRS: 3.88 ± 0.81; TDTmm: 0.6 ± 0.3; TDTnr: 0.9 ± 0.8; DSST: 21.5 ± 7.1;	NRS: 4.6 ± 1.7; TDTmm: 0.7 ± 1.1; TDTnr: 0.7 ± 0.62; DSST: 23.6 ± 7.2;	NRS: 0.97 ± 0.72; TDTmm: 0.7 ± 0.2; TDTnr: 8 ± 0.5; DSST: 22.4 ± 6.5;	In outpatient surgery, oral administration of *Passiflora* as premeditation reduces anxiety without sedation.
Ngan and Conduit, 2011 (Australia) [18]	Placebo	NA	NA	NA	NA	Consumption of a small dose of *Passiflora* infusion brings short-term subjective benefits to healthy adults with mild fluctuations in sleep quality.
Rokhtabnak et al., 2017 (Iran) [19]	Placebo	DSST: 30.67 ± nd; Ramsy scale: 1.81 ± nd; VAS: 26.5 ± nd;	DSST: 27.5 ± nd; Ramsey scale: 1.95 ± nd; VAS: 26.5 ± nd;	DSST: 22.33 ± nd; Ramsey scale: 1.85 ± nd; VAS: 26.5 ± nd;	DSST: 25.5 ± nd; Ramsey scale: 1.95 ± nd; VAS: 26.5 ± nd;	*Passiflora* premedication reduces anxiety, as does melatonin, but melatonin causes less cognitive disorders compared to *Passiflora*.

VAS (Visual Analogue Scale), NRS (Numerical Rating Scale), OAA/S (Observers Assessment of Alertness and Sedation Scale), DAS-R (Corah’s Dental Anxiety Scale, Revised), DSST (Digit Symbol Substitution Test), CPT (Continuous Performance Task/Test), TDT (Trieger Dot Test)*,* PAT (Perceptive Accuracy Test), FTT (Finger Tapping Test), STAI (State-Trait Anxiety Inventory), NA—not available, nd—no data

**Table 3:** Results of the systematic review. The following table refers to the results of the cross-review [15].

**What Did You Feel during the Surgery?**	**Protocol 1 (Midazolam)**		**Protocol 2 (*Passiflora*)**		**Results**
	**Midazolam (1)**	***Passiflora* (2)**	***Passiflora* (2)**	**Midazolam (1)**	*Passiflora* showed an anti-anxiety effect similar to midazolam; it was safe and effective in the case of conscious sedation in adult patients having their third mandibular molar tooth extracted
Calm	5 (33.3%)	17 (68%)	13 (52%)	3 (20%)	
Slight anxiety	6 (40%)	7 (28%)	10 (40%)	8 (55.3%)	
Serious anxiety or fear	3 (20%)	1 (4%)	2 (8%)	3 (20%)	
Bad feeling caused by anxiety	1 (6.7%)	0	0	1 (6.7%)	
Total	15	25	25	15	

**Table 4:** Risk of bias (ROB).

Reference/Country	Publication Year	Random Generation of The Error Sequence (Selection Error)	Hiding the Allocation (Selection Variation)	Blinding of Participants and Staff (Biased Evaluation)	Performance Evaluation Blindness (Detection Error)	Incomplete Result Data	Selective Reporting (Reporting Error)	Other Biases	Number of Indications with Low Risk of Bias
Akhondzadeh et al. (Iran) [12]	2001	L	?	L	?	L	L	L	5
Aslanargun et al. (Turkey) [13]	2012	?	?	L	?	L	L	L	4
Azimaraghi et al. (Iran) [14]	2017	L	L	L	L	L	L	L	7
Dantas et al. (Brazil) [15]	2017	L	L	L	L	L	L	L	7
Dimpfel et al. (Germany) [16]	2011	L	?	?	?	L	L	L	4
Kaviani et al. (Iran) [17]	2013	L	?	H	?	L	L	L	4
Movafegh et al. (Iran) [6]	2008	L	L	L	L	L	L	L	7
Ngan and Conduit (Australia) [18]	2011	?	L	L	L	L	L	L	6
Rokhtabnaket al. (Iran) [19]	2017	L	L	L	L	L	L	L	7

L—low risk of bias; H—high risk of bias; ?—unclear risk of bias.
